# Cosmetic Remodeling of the Smile: Combining Composite Resin and Ceramics over Teeth and Implants

**DOI:** 10.1155/2017/8698010

**Published:** 2017-08-03

**Authors:** Leonardo Fernandes da Cunha, Ubiracy Gaião, Rafael Coutinho Silva, Carla Castiglia Gonzaga, Gisele Maria Correr

**Affiliations:** Graduate Program in Dentistry, Universidade Positivo, Curitiba, PR, Brazil

## Abstract

The aim of this paper is to describe a restorative approach to the cosmetic remodeling of the teeth of a young adult patient with right maxillary lateral hypodontia and left lateral microdontia. A conservative restorative management was proposed to improve smile esthetics by combining direct composite resins and ceramics. Initially, periodontal therapy and dental bleaching were performed. Subsequently, direct composite resins were applied to the central incisors and canines to reestablish the sizes and shapes of these teeth. Finally, ceramics were placed on the implant and the microdontia to unite with the new alignment and color of the anterior teeth. Thus, conservative remodeling to improve the harmony of the smile was provided.

## 1. Introduction

Dental agenesis is a phenomenon that occurs with relative frequency. The incidence of missing teeth reported in the literature varies according to the population studied [1]. In some cases of lateral incisor agenesis, a microdontic tooth may also occur in its homologous form, that is, with a smaller size. The etiology of agenesis is unknown; however, a family tendency has been reported [2]. In the primary dentition there is no significant gender-based distribution, but in the permanent dentition women are more frequently affected than men by a proportion of 3 : 2 [3]. Cases of agenesis and microdontic teeth are a challenge for the clinician.

In many cases, a combined orthodontic-restorative-surgical approach is used to redistribute space in preparation for future implants or prostheses [4, 5]. These cases will always require maintenance and future replacements. The replacements for these restorations must be made with careful planning so that they present the greatest possible longevity. The improvement of restorative techniques and materials used to satisfy this market demand has created an increase in treatment options that can improve smile harmony with a conservative approach.

Extremely thin dental ceramics reinforced with lithium disilicate have been widely recommended for the treatment of microdontic teeth and diastemata [6], presenting esthetic excellence and being as conservative as composite resins. The same ceramics can be used on implant prostheses. However, certain precautions are specific to this kind of treatment, such as advance planning, the maximum number of sessions, and the manufacture of temporary restorations.

To favor the harmony of the smile, tooth remodeling can be done with composite resin. The direct adhesive restorative system provides excellent esthetic and functional results, as well as presenting lower cost and clinical time relative to that required for indirect treatments, making it a viable alternative for both patient and clinician [7]. In addition, the resins can be associated with previous dental bleaching to favor the color of the substrate [8]. Therefore, the objective of the present work was to present the smile “reshaping” of a patient with lateral agenesis and microdontic teeth, using the application of periodontal surgery procedures, composite resin and ceramics.

## 2. Case Report

**Figure 1:**
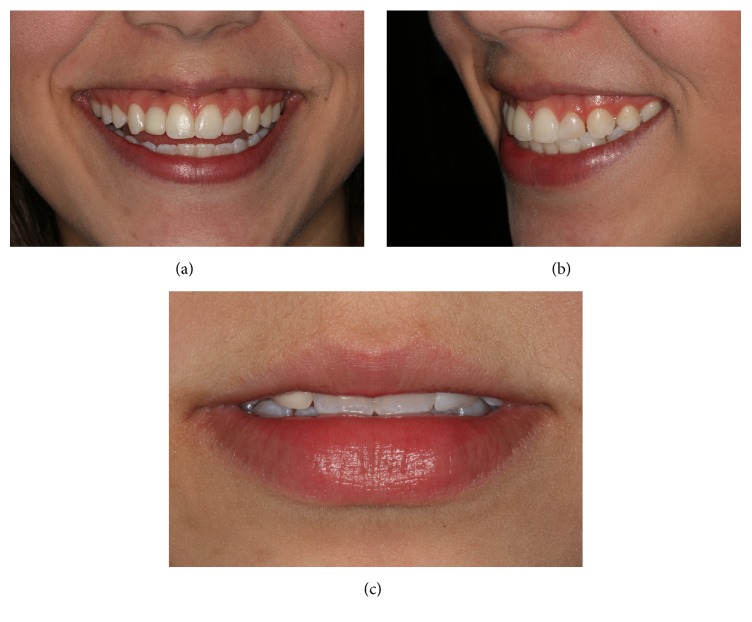
Initial appearance of the patient's smile from the front (a) and lateral (b) perspectives. She was dissatisfied with the shapes, sizes, and color of her teeth. Lips at rest show the excessive opacity of the prosthesis on the right side lateral implant (c).

**Figure 2:**
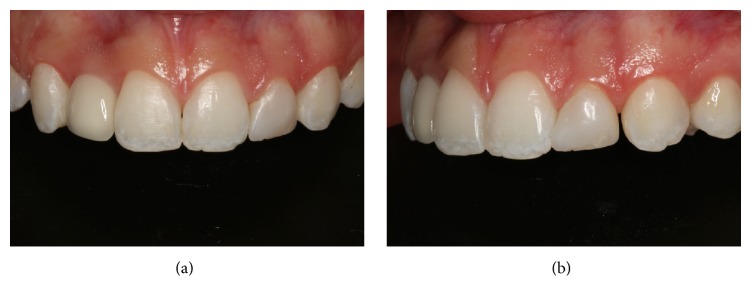
Detail of anterior teeth, showing imbalance of maxillary gingival arch harmony and unsatisfactory resin restoration on the left side lateral incisor. Front (a) and left (b) details of the maxillary anterior teeth.

**Figure 3:**
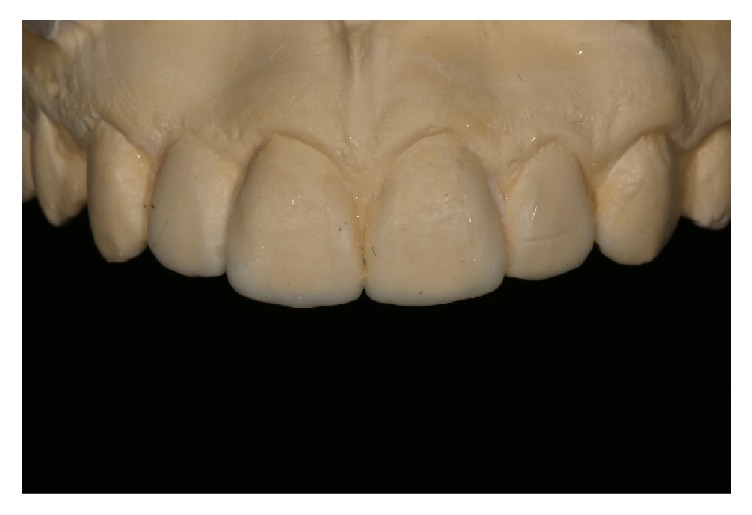
Model of diagnostic wax-up. Note the more defined and triangular contour of the buccal proximal edges.

A young adult female patient (20-year-old) attended the clinic of restorative dentistry specialization course, complaining of dissatisfaction with the shape, staining, and fractures of composite resin restorations. The patient's history showed no extractions, previous orthodontic treatment, or implants. Clinical examination revealed a composite resin on the left lateral microdontic incisor, a prosthetic implant on the right lateral incisor, and hypoplastic stains on the central incisors and maxillary and mandibular canines. In addition, the gingival concave arch was irregular but gingival inflammation was not observed. The composite resin restorations of lateral incisors also were not maintaining adequate width-to-height ratio. Radiographic examination confirmed the presence of the implant and the health of adjacent tissues (Figures 1 and 2). Models and photographs were prepared for case study. A diagnostic wax-up was done on the maxillary arch model (Figure 3).

**Figure 4:**
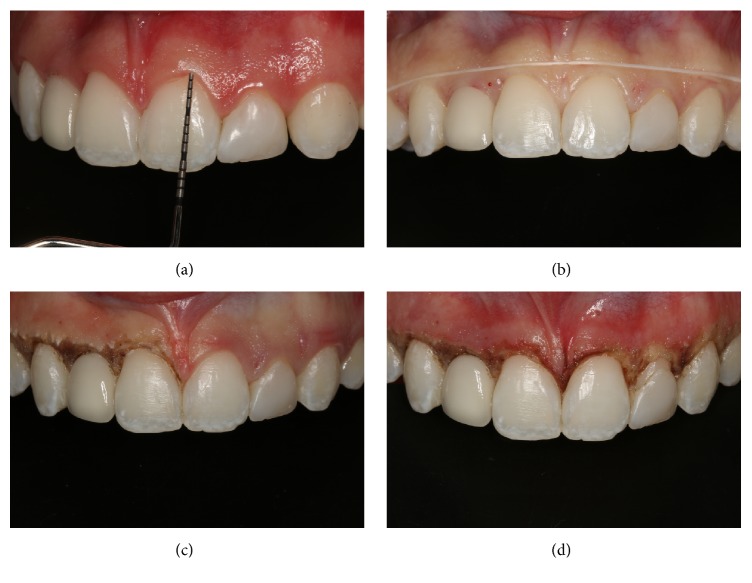
Probing after the scaling and prophylaxis sessions, performed prior to the surgical treatment (a). The smile line was marked with dental floss (b). Gingival recontour with an electric scalpel on the right side (c). Appearance immediately after gingival recontour (d).

Initial prophylaxis (CleanJoy, Cuxhaven, Germany) and scaling of the teeth were indicated prior to the surgical procedure. Despite the patient's periodontal health, it was still necessary to reconstruct gingival harmony. After four weeks, surgery was performed to increase the clinical crowns of the teeth. Probing with a millimeter probe was performed, and surgery was performed with an electric scalpel (Deltronix Bo 1300, Ribeirão Preto, São Paulo, Brazil), without the need for bone contouring. Gingivectomy was performed to achieve the desired gingival margin position, thus, improving the width-to-height ratio of dental crowns. Sutures were not needed. Following the surgical procedure, oral diclofenac sodium 100 mg was administered for 3 days. Dental prophylaxis was achieved 30, 60, and 90 days after surgery. Postoperative follow-up was done after 3 months (Figure 4).

**Figure 5:**
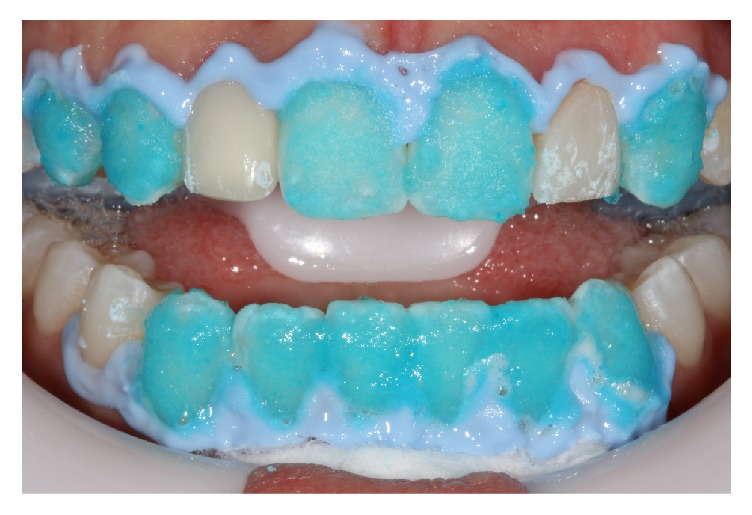
In-office bleaching with hydrogen peroxide.

Dental bleaching was then performed in the dental office. A labial retractor (Arcoflex, FGM, Joinville, Brazil) was added to improve visibility and protection of soft tissues. The gingival barrier (Polaoffice Powder, SDI, Victoria, Australia) was applied and photopolymerized according to the manufacturer's recommendation. A buccal mirror was used to determine whether there were regions lacking the barrier, to avoid damage to the soft tissues. The 35% hydrogen peroxide bleaching gel kit (Polaoffice Powder, SDI, Victoria, Australia) was applied according to the manufacturer's instructions. The powder and liquid were mixed well to be homogenized in a gel of suitable viscosity. With the aid of a microapplicator, the product was placed on the buccal surfaces of the teeth, except for the maxillary lateral incisors that had restorations. The application was done without the addition of light. At the end, the gel was removed with suction and the aid of a piece of gauze. The teeth were washed, and this procedure was repeated two more times; that is, the gel was applied 3 times in that session. Four weeks of home bleaching with 10% carbamide peroxide was recommended (Pola Night, SDI, Victoria, Australia) (Figure 5).

After two weeks for the color to stabilize, the final color was selected. The absolute isolation of the operative field was performed by means of a Hygienic rubber dam sheet (Coltene/Whaledent AG, Altstätten, Switzerland). Polytetrafluoroethylene (TDV, Pomerode, Santa Catarina, Brazil) tape was initially placed on the lateral incisors to prevent acid conditioning of these teeth.

The enamel surfaces of the central incisors and canines were conditioned with phosphoric acid over the teeth to avoid the application of resin over unconditioned areas. The surfaces were then dried, the adhesive (Futurabond, VOCO) was applied by means of a regular-sized microbrush (Coltene/Whaledent AG, Altstätten, Switzerland), and polymerization was carried out according to the manufacturer's instructions. Amaris resin in the TL color (Voco, Cuxhaven, Germany) was applied freehand, that is, without the aid of a silicone wall, with the aid of a CompoRoller spatula (Kerr, Orange, CA, USA). Only this resin color was used to reshape the teeth and cover the hypoplastic stains. Each increment was polymerized at the time recommended by the manufacturer, continuously and as closely as possible to the material without, however, touching the resin. In a subsequent session, the finishing and polishing of the resins were done with the Super Tray (Kerr, Orange, CA, USA) kit.

**Figure 6:**
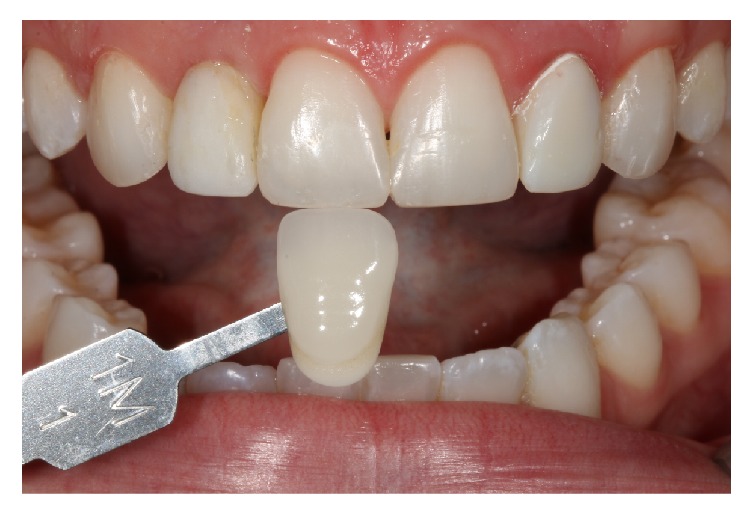
After recontouring with composite resin (Amaris) and provisional restoration with bisacrylic resin (Structur 3), the ceramic color was selected from the VITA 3D Master scale.

**Figure 7:**
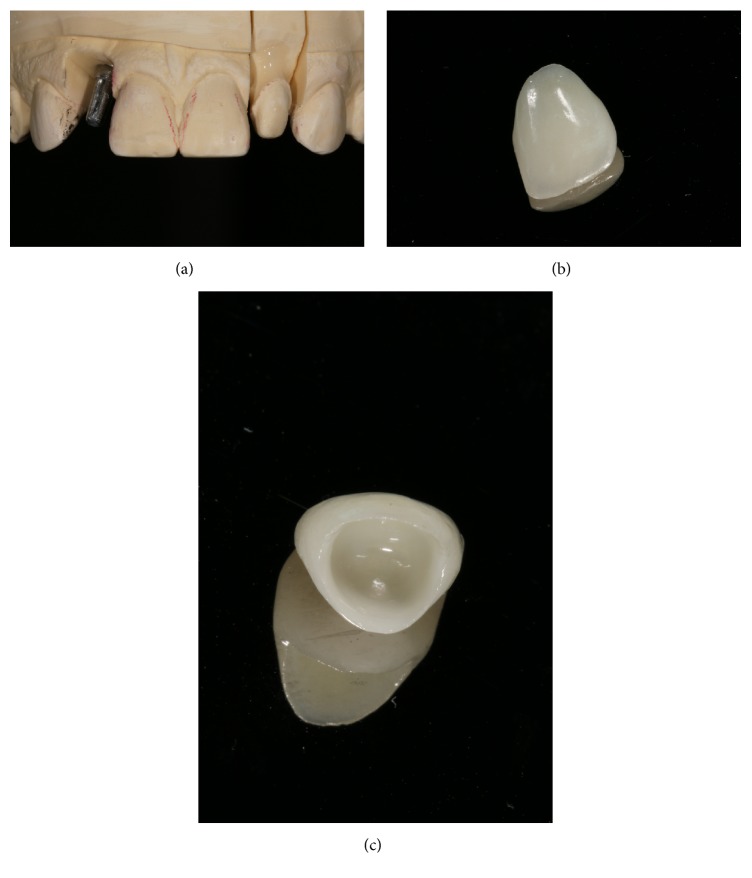
Model (a) with die. Reinforced ceramic with lithium disilicate in the BL4 color. External and internal aspects of ceramics on the microdont tooth after finishing (b and c).

Temporary restorations with bisacrylic resin (Structur 3, Voco, Cuxhaven, Germany) were made on the lateral incisors (Figure 6). The molding was done with the double-wire technique on the lateral microdontia (Sure-Cord, Sure Dent Corporation, Gyeonggi-do, Korea) simultaneously with the addition of reaction silicone (Variotime, Heraeus Kulzer, Hanau, Germany). The ceramic was prepared with the E.max injected system (Ivoclar Vivadent, Schaan, Liechtenstein) in the color BL4 and applied to the ceramic for intrinsic incisal characterization (Figure 7).

**Figure 8:**
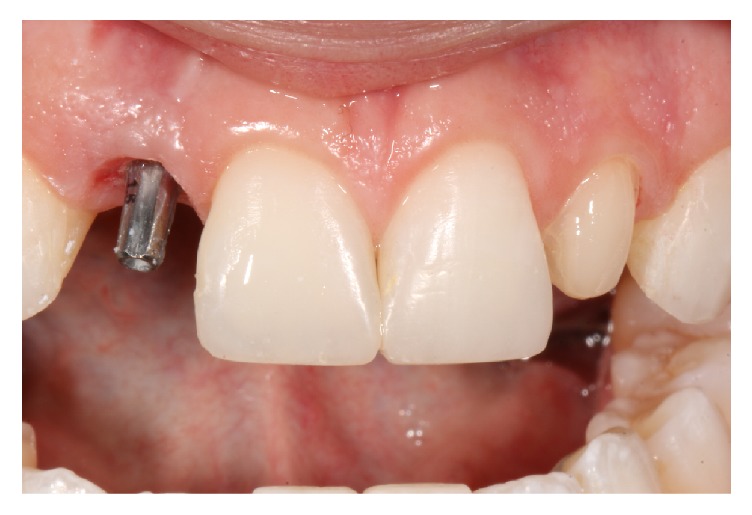
Removal of temporary restorations. Note the absence of soft tissue inflammation, demonstrating adequate adaptation of the provisional restorations with bisacrylic resin.

The material used for the cementation of the ceramics on the lateral incisors was Bifix SE self-adhesive cement (Voco, Cuxhaven, Germany) in opaque white (WO) (Figure 8). Prophylaxis with prophylactic paste was done prior to cementation. Neighboring teeth were protected with a Teflon strip (TDV, Santa Catarina, Brazil). The self-adhesive cement selected was inserted inside the restorations and placed in position. Excesses were removed with dental floss and a microbrush, and the cement was polymerized as recommended by the manufacturer.

**Figure 9:**
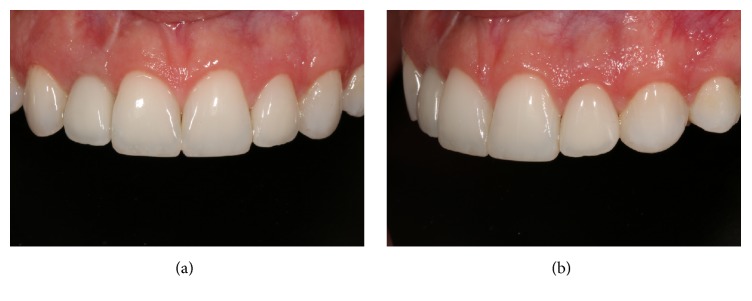
Front (a) and left (b) detail of resin restorations combined with ceramics.

**Figure 10:**
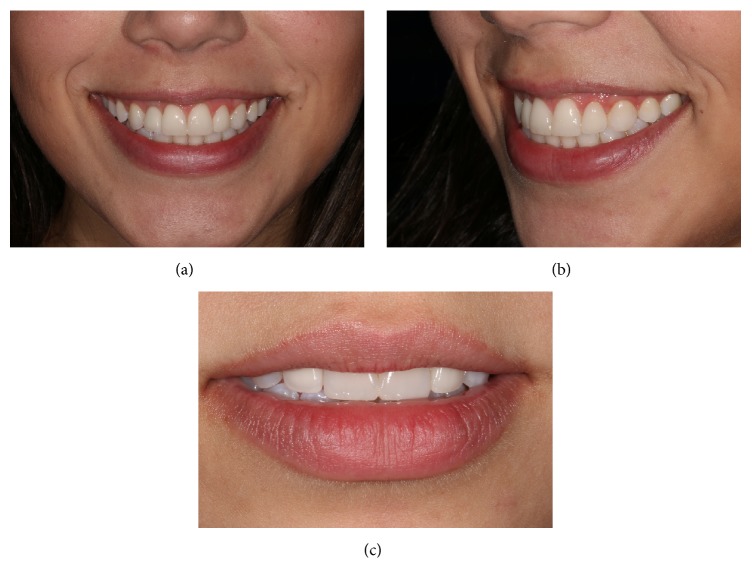
Frontal and lateral views of the patient's final smile. Final view of the teeth with resting lip, demonstrating improved translucency of the prosthesis over the implant.

After cementation, the occlusal contacts were checked and adjusted. The results can be seen in Figures 9 and 10.

## 3. Discussion

Lateral agenesis is relatively frequent in the population [1]. An integrated treatment approach with orthodontics-implants-restorations is often used [2, 3]. Thus, the clinician should know not only how to guide the treatment, but also how to follow it in the long term in the best way possible.

Gingival contour is fundamental for smile esthetics. Different surgical procedures are available for the adequate management of periodontal soft tissues with the aim of providing esthetic patterns. Most recently, focus has been on the clinical crown increases of the anterior-superior teeth, at the expense of gingival tissue only, without involvement of bone tissue [9]. An electric scalpel can be used for ease, speed, and obtaining the desired results.

In the presented case, the central incisors and canines showed hypoplasia and irregular anatomy. Composite resin was applied to the buccal surfaces of these teeth to modify the anatomy and color. The incisal areas of the central incisors and canines were enlarged, and the edges were better-defined. The direct adhesive restorative system used provided minimal wear of tooth structure, good clinical durability, and low cost. Further, the treatment is reversible and gives excellent esthetics [7].

Historically, tooth whitening has been a widely used conservative alternative for improving tooth color, and different methods have been used, with generally good esthetic results. In the case presented, both in-office and home whitening procedures were performed. In addition, tooth whitening has the advantage of allowing for association with other esthetic and/or cosmetic restorative techniques, as shown here [6–8].

In cases of single implant prostheses used on anterior teeth, it is always an esthetic challenge to match these restorations with the other teeth. In the case presented, the initial image showed the higher opacity of this restoration. To favor esthetics, a restoration with a zirconia structure was used to mask the metal and to facilitate the characterization of the overlying ceramic. The esthetics and longevity of the E.max system were widely reported in the literature, supporting its choice for the case in question [6, 10].

## 4. Conclusion 

Thus, the association of composite resins and ceramics after periodontal treatment and tooth whitening for remodeling a smile is a conservative, efficient, and safe approach to esthetic improvement.
